# Taxonomic and Functional Diversity Provides Insight into Microbial Pathways and Stress Responses in the Saline Qinghai Lake, China

**DOI:** 10.1371/journal.pone.0111681

**Published:** 2014-11-03

**Authors:** Qiuyuan Huang, Brandon R. Briggs, Hailiang Dong, Hongchen Jiang, Geng Wu, Christian Edwardson, Iwijn De Vlaminck, Stephen Quake

**Affiliations:** 1 Department of Geology and Environmental Earth Science, Miami University, Oxford, Ohio, United States of America; 2 State Key Laboratory of Biogeology and Environmental Geology, China University of Geosciences, Beijing, China; 3 State Key Laboratory of Biogeology and Environmental Geology, China University of Geosciences, Wuhan, China; 4 Department of Microbiology, University of Georgia, Athens, Georgia, United States of America; 5 Departments of Bioengineering and Applied Physics, Stanford University and the Howard Hughes Medical Institute, Stanford, California, United States of America; CAS, China

## Abstract

Microbe-mediated biogeochemical cycles contribute to the global climate system and have sensitive responses and feedbacks to environmental stress caused by climate change. Yet, little is known about the effects of microbial biodiversity (i.e., taxonmic and functional diversity) on biogeochemical cycles in ecosytems that are highly sensitive to climate change. One such sensitive ecosystem is Qinghai Lake, a high-elevation (3196 m) saline (1.4%) lake located on the Tibetan Plateau, China. This study provides baseline information on the microbial taxonomic and functional diversity as well as the associated stress response genes. Illumina metagenomic and metatranscriptomic datasets were generated from lake water samples collected at two sites (B and E). Autotrophic *Cyanobacteria* dominated the DNA samples, while heterotrophic *Proteobacteria* dominated the RNA samples at both sites. Photoheterotrophic *Loktanella* was also present at both sites. Photosystem II was the most active pathway at site B; while, oxidative phosphorylation was most active at site E. Organisms that expressed photosystem II or oxidative phosphorylation also expressed genes involved in photoprotection and oxidative stress, respectively. Assimilatory pathways associated with the nitrogen cycle were dominant at both sites. Results also indicate a positive relationship between functional diversity and the number of stress response genes. This study provides insight into the stress resilience of microbial metabolic pathways supported by greater taxonomic diversity, which may affect the microbial community response to climate change.

## Introduction

Microorganisms have been key respondents to and drivers of global climate change by affecting the atmospheric concentrations of greenhouse gases [1]. The microbial response to future global climate change is likely controlled by the biodiversity (i.e. taxonomic and functional diversity) of the ecosystem [2], [3], [4]. For example, ecosystems with higher biodiversity are more likely to be stable against environmental change because of a greater likelihood of having key functioning species [5]. However, ecosystems with low diversity can be stable if the organisms have mechanisms to respond to stress [6]. For example, *Synechococcus* has multiple protective mechanisms to cope with UV stress and can maintain photosynthesis, while *Procholorcoccus* lacks these protective mechanisms and shuts down several key metabolic processes under similar UV stress [7]. Thus, studying the biodiversity and potential stress response mechanisms can aid in understanding microbial community response to stress.

Baseline information on the microbial biodiversity and stress response mechanisms is needed in ecosystems that are sensitive to climate change. For example, the Tibetan Plateau has experienced significant warming in recent decades and is considered to be a sensitive indicator of regional and global climate change [8]. Temperature has increased 0.28°C per decade since the early 1960 s [9] causing 82% of the 46,000 glaciers to retreat [10]. The melting glaciers have caused numerous floods and altered salinity and water levels in most of the Tibetan lakes [11]. The fragility and sensitivity of the Tibetan Plateau’s ecosystem to these environmental changes have resulted in loss of habitats and extinctions of endemic macrobiota [12].

Qinghai Lake, located on the Tibetan Plateau, is characterzied by oligotrophy, low temperature, moderate salinity, and high UV radiation, making it a unique ecosystem for studying microbial response to global climate change [13]–[15]. Previous studies on Qinghai Lake have detected novel archaea commonly found in marine environments [16], and the microbial diversity, composition, and lipid profiles all showed a response to salinity change [14], [17], [18]. However, the following key questions remain unanswered: (1) what is the microbial taxonomic and functional diversity for the Qinghai Lake water column, (2) what is the metabolic potential and active metabolisms related to the carbon and nitrogen cycles, and (3) what stress response genes are present in organisms involved in the carbon and nitrogen cycles? Answering these questions can provide baseline knowledge that can be used to understand the effect of biodiversity on the microbial community response to environmental stress.

An integrated approach including geochemical, metagenomic and metatranscriptomic analyses were used to answer these questions. The metagenomic and metatranscriptomic reads were annotated with both taxonomic and functional information. The synthesized cDNA was compared to the DNA retrieved from the same sample to assess the relative activity of different populations and functional gene transcription in the microbial community. In addition, the relationship between species richness and the number of stress response genes was assessed. Microbial processes in Qinghai Lake are involved in both the carbon and nitrogen biogeochemical cycles. However, certain processes (e.g., photosynthesis, denitrification) had lower diversity and fewer stress response genes, which may make them more susceptible to environmental stresses.

## Materials and Methods

### Site description

**Figure 1:**
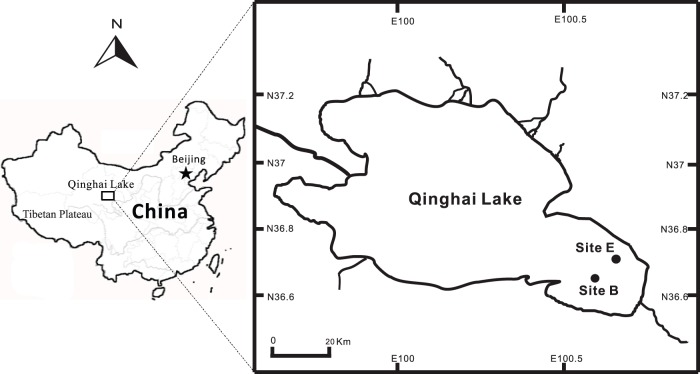
A geographical map showing the sampling sites in Qinghai Lake, China.

Qinghai Lake is a perennial lake located on the Tibetan Plateau at an elevation of 3196 m above sea level. The lake is located in a structural intermontane depression at the north-eastern corner of the Qinghai-Tibetan Plateau (Figure 1) [19]. The lake has a surface area of 4300 km^2^ and lies within a catchment of limestone, sandstone, and shale. The average water depth is 19.2 m with the maximum of 28.7 m. The evaporation of the lake (∼1400 mm/year) is in excess of mean annual precipitation (∼400 mm/year), resulting in a mesohaline lake. Qinghai Lake is separated into two subbasins by a normal faulting horst in the middle of the lake. The northern subbasin is more dynamic than the southern subbasin because of riverine input. The southern subbasin water is stratified in winter due to ice cover, but in summer, the water column geochemistry is fairly uniform [14], [16]. No specific permits were required for the described field studies because no animal or human subjects were involved in this research. The sampling locations are not privately owned or protected in any way. The field studies did not involve endangered or protected species.

### Sampling and geochemical analysis

Water samples were collected from the southern subbasin mid-day in August 2011 at sites B and E (Figure 1). The water samples were pumped from water depths of 12.5 m and 13.6 m below the lake surface from sites B and E, respectively. These two water depths were selected as representatives of the microbial community in the water column. Ten to 12 L of lake water was filtered through a polyethersulfone filter with a pore size of 0.2 µm (Supor; Pall Life Sciences, Ann Arbor, MI, USA). A Horiba multi-parameter meter (W-20XD Series, HORIBA, Kyoto, Japan) was used to measure *in-situ* environmental parameters of the water including; temperature, pH, conductivity, dissolved oxygen (DO), depth, chloride (Cl^−^), and salinity at both sites. Concentrations of major ions (i.e., sulfide, sulfate, silica, nitrite, nitrate, ferrous iron, and ammonia) in the filtered water of both sites were measured using a HACH colorimeter (model CEL 850, HACH Chemical Co., Iowa, USA) as previously described [20]. Filters collected for microbial analysis were placed on dry ice immediately after filtering of lake water from each site, and stored at −80**°**C in the laboratory until DNA and RNA extractions were conducted. A portable global positioning system (GPS) unit (eTrex H, Garmin) was used to indicate the location of each sampling site (Figure 1).

### RNA and DNA extractions

Total RNA was extracted from one half of the filter from each site following a modified version of the RNeasy kit (Qiagen, Valencia, CA, USA) as previously described [21]. Briefly, half of each frozen filter was thawed and vortexed for 10 min with 8 ml of RLT lysis buffer and 3 g of low protein binding zirconium beads (200 µm, OPS Diagnostics, Lebanon, NJ, USA). RNA was then extracted using the RNeasy kit according to manufacturer’s instructions. A Turbo DNA-free kit (Ambion, Austin, TX, USA) was used to remove any residual DNA. The resulting RNA was purified and concentrated using the RNeasyMinElute Cleanup kit (Qiagen, Valencia, CA, USA) [22]. Genomic DNA was extracted from the other half of the filter using the FastDNA Spin Kit (MP Biomedical, OH, USA) as previously described [20]. The amount of DNA that was extracted from sites B and E was 14.6 and 13.8 ng µl^−1^, indicating that DNA was extracted from roughly the same amount of biomass.

### Metatranscriptomic sample preparation

Ribosomal RNA (rRNA) was removed from total RNA via a subtractive hybridization process using sample-specific biotinylated rRNA probes [23]. rRNA-subtracted RNA was amplified linearly using the MessageAmp II-Bacteria kit (Ambion, Austin, TX, USA). Amplified RNA was converted to double-stranded cDNA using the Universal RiboClone cDNA synthesis system (Promega, Madison, WI, USA) using random hexamer primers [23]. The synthesized cDNA was purified with the QIAquick PCR purification kit (Qiagen, Valencia, CA, USA).

### Library preparation and sequencing

The extracted DNA and synthesized cDNA was sheared to 500 base pairs (bp) using a Covaris ultrasonicator (Covaris Inc., Woburn, MA, USA) according to manufacturer’s recommendations. The sheared DNA was end-repaired, adaptor-ligated with multiplexing, and purified using the Ovation SP ultralow DR multiplex system (NuGEN Technologies Inc., CA, USA). The prepared library was then sequenced using an Illumina MiSeq (250 bp paired-end reads).

### Data analysis

Sequences were paired, trimmed, and filtered using the CLC Genomics Workbench version 6 (CLC Bio, Aarhus, Denmark). Paired reads were assembled together if 25 bp overlapped. Reads were trimmed based on the length (minimum length 50 bp) and quality (quality score ≥20) [24]. Sequences were uploaded to the metagenomics RAST (MG-RAST) server [25] for annotation and are available under MG-RAST ID 4532866.3, 4532865.3, 4522126.3, and 4522125.3 for the samples B_DNA, B_RNA, E_DNA, and E_RNA, respectively.

Sequences for DNA and RNA libraries were assigned a taxonomy and function using BLASTX [26] on MG-RAST v3.3.6 against M5NR database, which integrates multiple databases (e.g., NCBI-nr, KEGG, SEED, and *etc.*). Sequences were given an annotation if it had at least a bit score cut-off of 50, E-values of 1×10^−5^, and a minimum alignment of 15 amino acids [24]. DNA and RNA reads that were annotated with taxonomy and function were downloaded from MG-RAST and a custom R script was used to search for functional annotations involved in the carbon and nitrogen cycles, and stress responses [27]. KEGG categories were used to determine annotations that were involved in stress response. The richness of each functional gene was determined by counting the number of species or phyla that contained that particular functional gene.

Rarefaction curves were created for each of the samples to determine whether the sequencing depth was sufficient to detect the majority of species containing a functional gene. This was performed using the “rarecurve” function in the R package “Vegan” [28]. A network of correlated genes (i.e. functional genes found [DNA] or expressed [RNA] in the same organisms as stress response genes) was created using the WGCNA package [29] and the igraph package [30] in R [27], and viewed in Cytoscape [31]. Species with only one functional gene and functional genes in only a single organism were removed to reduce the complexity of the network [32]. A Spearman correlation between each functional gene and a stress response gene was calculated with a p-value adjusted for multiple comparisons. Correlations with a p-value greater than 0.05 were removed from the network.

### *amoA* amplification

Jiang et al. [19] detected abundant ammonia oxidizing bacteria (AOB) and ammonia oxidizing archaea (AOA) in Qinghai Lake; however, the ammonia monooxygenase (*amoA)* gene was not detected in our metagenomic or metatranscriptomic datasets (see results below). Therefore, the *amoA* gene was amplified from the same DNA pool used for metagenomics. PCR amplifications were performed using FailSafe PCR System (Epicentre Biotechnologies, Madison, WI), AOB specific primer set: amoA-1F (5′-GGGGTTTCTACTGGTGGT-3′) and amoA-2R (5′-CCCCTCKGSAAAGCCTTCTTC −3′), and with the same conditions as described previously [33]. The amplicons were stained with EtBr and visualized on a 1% agarose gel.

## Results and Discussion

### Water geochemistry

Sites B and E shared similar geochemical profiles for most parameters (Table 1). For example, both sites had 1.4% salinity and a pH of ∼9.1. The DO content was 8.9 and 8.7 ppm for sites B and E, respectively. Site E was about 1 m deeper and 2**°**C warmer and had a higher ammonia concentration (1 mg/L) than site B (below detection).

**Table 1:** Sample locations and geochemistry.

Parameters	B	E
GPS location (N, E)	36.66, 100.60	36.74, 100.69
Temperature (°C)	13.7	15.6
pH	9.1	9.2
Depth (m)	12.5	13.6
Conductivity (s/m)	2.28	2.26
DO (ppm)	8.9	8.7
Salinity (%)	1.4	1.4
Cl^−^ (mg/L)	2930	3420
NH_4_^+^ (mg/L)	ND	1
NO_3_^−^ (mg/L)	0.39	0.4
NO_2_^−^ (mg/L)	0.13	NA
Si (mg/L)	1.43	NA
S^2−^ (mg/L)	0.05	ND
SO_4_^−^ (mg/L)	>80	>80
Fe^2+^ (mg/L)	0.065	NA

ND: not detected. NA: not available.

### Descriptive statistics of DNA and RNA

After quality control of sequence reads, a total of 10,514,407 and 4,035,731 reads were retrieved in the DNA libraries for sites B and E, respectively (Table 2). The RNA libraries had a total of 1,728,111 and 2,893,577 reads for sites B and E, respectively (Table 2). Twenty-eight percent to 81% of the DNA and RNA reads that were identified to have an open reading frame were annotated with both function and organism (Table 2). Protein and rRNA features were predicted and identified for RNA and DNA libraries from both sample sites (Table 2).

**Table 2:** Statistic results of metagenome and metatranscriptome sequences from sites B and E in Qinghai Lake.

	B		E	
	RNA	DNA	RNA	DNA
Number of reads	1,728,111	10,514,407	2,893,577	4,035,731
Mean sequence length (bp)	211±60	194±55	200±59	185±59
Total Mbp	365	2,049	581	749
Mean GC content (%)	49±8	60±8	44±9	63±5
Reads with ORF^a^	1,463,714	10,161,795	2,098,251	3,901,755
Identified protein features	288,106	3,233,826	304,159	945,021
Identified rRNA features	413,195	10,573	113,464	2,307
% annotated reads^b^	65.0	81.2	28.3	77.3

a Open reading frame.

b % of reads with an open reading frame that were annotated by function and taxonomy.

base pairs (bp).

### Taxonomic diversity

Protein-coding genes, while not as phylogenetically robust as 16S rRNA genes, can be used to identify approximate taxonomic affiliations [34], [35]. A total of 832 genera within 31 phyla were detected. The DNA samples from both sites contained more genera than were detected in the RNA samples, indicating some genera had no or low activities (Table 2). Bacterial sequences dominated both locations, with each sample containing fewer than 1% archaeal sequences. Eukaryotic sequences were removed for further analysis because of the unreliability of FragGeneScan (prokaryotic gene calling algorithm used by MG-RAST) to identify eukaryotic open reading frames [36] and our hypotheses were specific to prokaryotes.

**Figure 2:**
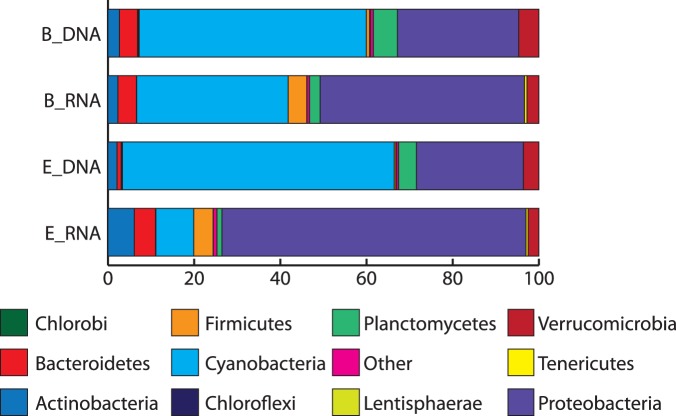
Distribution of phyla detected in the DNA or RNA samples from sites B and E determined by taxonomic assignment of metagenomic or metatranscriptomic reads. Phyla with <1% abundance were grouped into “other”.

At the phylum level, *Cyanobacteria* dominated the B_DNA and E_DNA samples with 52% and 63% of the total abundance, respectively (Figure 2). *Synechococcus* was the dominant genus, which has been previously detected in clone libraries from Qinghai Lake [14], [15], [37] and is known to be important contributors to carbon fixation in freshwater and marine ecosystems [38], [39]. The second most abundant phylum in the DNA samples was *Proteobacteria,* with 28 and 24% of total abundance in B_DNA and E_DNA, respectively (Figure 2). Within the *Proteobacteria*, *Loktanella* was the dominant genus. It is an aerobic heterotroph that can supplement its energy requirements through aerobic anoxygenic photosynthesis [40], [41]. Other major phyla that were detected in the DNA samples included *Actinobacteria, Bacteroidetes, Planctomycetes*, and *Verrucomicrobia* (Figure 2).

In the RNA samples, *Proteobacteria* were the most abundant (B_RNA: 47%, E_RNA: 70%) and *Cyanobacteria* were the second most abundant (B_RNA: 35%, E_RNA: 8%) (Figure 2). Other major phyla detected in the RNA samples were *Firmicutes*, *Actinobacteria*, *Bacteroidetes*, and *Verrucomicrobia* (Figure 2). Differences in RNA degradation rates and possible mis-annotations make it difficult to access true activity levels, but the relative activity levels can be defined as the ratio of RNA to DNA [42]. With this assumption, *Proteobacteria* were 1.6 and 2.8 fold more active than *Cyanobacteria* in samples B_RNA and E_RNA, respectively.

### Metabolic Pathways in the Carbon Cycle

**Figure 3:**
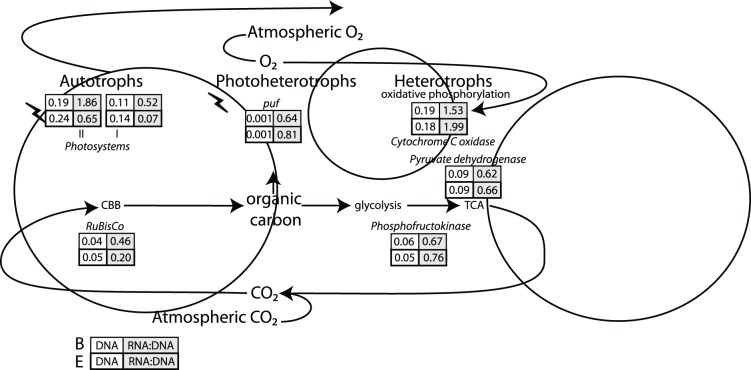
The carbon cycle depicted by a generalized autotroph, photohetertroph, and heterotroph in Qinghai Lake. The numbers in boxes represent the percentage and the RNA:DNA ratio of reads that were annotated within each metabolic pathway for sites B and E. The key genes used to identify a pathway was Ribulose-bisphosphate carboxylase (RuBisCo): Calvin-Benson-Bassham cycle (CBB), D-glucose 6-phosphotransferase: glycolysis, pyruvate dehydrogenase: tricarboxylic acid cycle (TCA), and cytochrome C oxidase: oxidative phosphorylation.

Functional identification of protein-coding genes identified autotrophic and heterotrophic carbon metabolisms (Figure 3). At both sites carbon fixation was performed by ribulose bis-phosphate carboxylase (RuBisCo), which is indicative of the Calvin-Benson-Bassham (CBB) cycle [43]. However, activity levels of RuBisCo (RNA:DNA 0.46 [B] and 0.2 [E]) were low compared to heterotrophic processes (see below).

**Figure 4:**
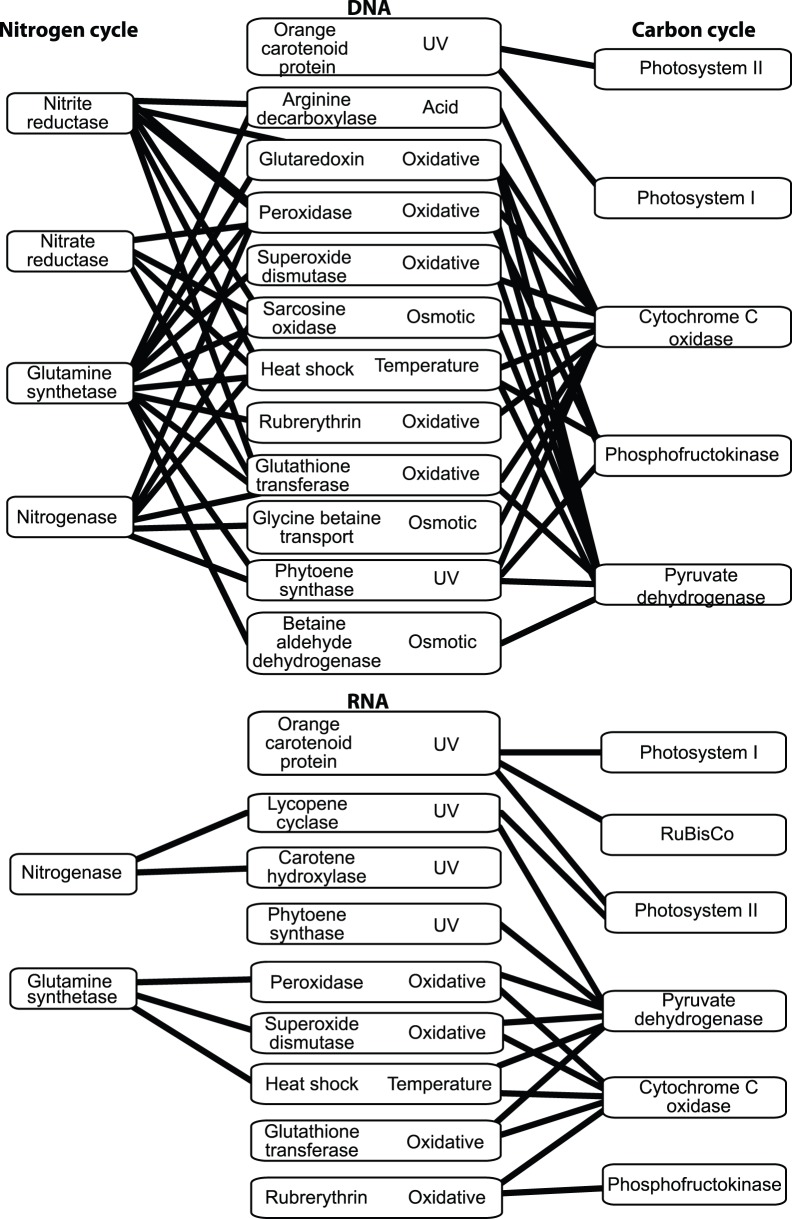
Network showing correlations of carbon and nitrogen cycle-related genes with stress response genes for DNA and RNA samples. Lines represent genes that are directly correlated (i.e. detected (DNA) or expressed (RNA) in the same species). Only significant correlations (p<0.05) are shown. The type of stress that each gene is involved in is also listed.

Photosystems I and II were detected, indicating the reliance of carbon fixation on light energy (Figure 3). Photosystem II (RNA:DNA 1.86 [B] and 0.65 [E]) was more highly expressed than photosystem I (RNA:DNA 0.52 [B] and 0.07 [E]) (Figure 3). The higher expression of photosystem II than photosystem I indicate that the *Cyanobacteria* are under UV stress [44], which is expected in Qinghai Lake because of its high UV irradiance due to the high elevation. In addition, network analysis revealed that organisms expressing photosystems I and II were also expressing orange carotenoid protein (Figure 4), which plays an important role in protecting photosynthetic organisms (such as *Cyanobacteria*) from solar radiation [45], [46]. These data suggested that the photoautotrophs in Qinghai Lake had low activities and were under UV stress.

Heterotrophic remineralization of organic carbon proceeded through the glycolysis and tricarboxylic acid cycle (TCA). The key enzymes for glycolysis (phosphofructokinase- RNA:DNA 0.67 [B] and 0.76 [E]) and the TCA cycle (pyruvate dehydrogenase- RNA:DNA 0.62 [B] and 0.66 [E]) had slightly higher RNA:DNA ratios than RuBisCo. Oxidative phosphorylation, identified by cytochrome C oxidase, was the most abundant and active (RNA:DNA 1.53 [B] and 1.99 [E]) energetic pathway. Aerobic anoxygenic phototrophic *puf* operon was also detected indicating that some energy generation came from photoheterotrophy (RNA:DNA 0.64 [B] and 0.81 [E]). The high concentration of DO (8.7–8.9 ppm) suggests aerobic respiration was the most prominent. Network analysis showed that these heterotrophs were also expressing genes involved in osmotic and oxidative stress (Figure 4), again consistent with the saline and oxic nature of the water in the lake.

### Metabolic Pathways in the Nitrogen Cycle

**Figure 5:**
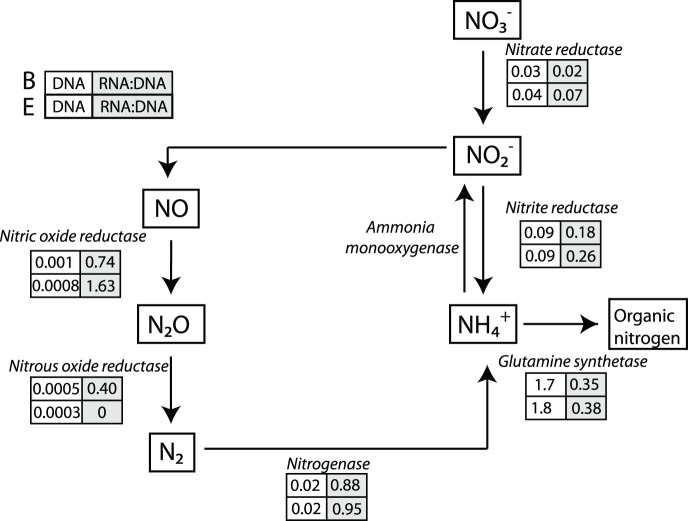
DNA and RNA reads detected and annotated within the nitrogen cycle. The numbers in boxes represent either the percent or the RNA:DNA ratio of reads that were annotated as genes within each pathway for sites B and E.

Nitrogen is biologically available in the form of ammonia. Ammonia assimilation into organic molecules occurred primarily via the glutamine synthetase pathway (Figure 5). The high functional activity of the ammonia assimilatory pathway observed probably led to the low concentration of ammonia measured at both sites (≤1 mg/L). Active nitrogen fixation genes were also detected, which is possibly driven by low levels of ammonia in the lake (Figure 5). Denitrification pathways (i.e., nitric oxide reductase and nitrous oxide reductase) had very low abundance but were highly transcriptionally active (Figure 5). Denitrification requires anoxic conditions, so it was unexpected to detect denitrification transcripts in the oxic water column. It is possible that localized anoxic conditions can exist because of oxygen micro-gradients, shown in marine snow [48] and microbial mats [49]. These small patches of anoxic water could harbor a relatively low abundance of active anaerobic denitrifiers.

Biological ammonia oxidation is the first step in the nitrification process (NH_3_ → NO_2_^−^) and is mainly carried out by AOB and AOA that contain the *amoA* gene [50]. Putative AOB and AOA were detected at both sites. AOB genera that were detected included *Nitrosospira*, *Nitrosomonas* and *Nitrosococcus* and AOA genera that were detected included *Nitrosocaldus* and *Nitrosopumilus*. The AOB were more abundant than AOA in all samples; however, AOB and AOA comprised less than 0.1% and 0.01% of total reads, respectively. Although putative AOB- and AOA-like sequences were identified, the *amoA* gene was not detected in our metagenomic or metatranscriptomic samples. However, the *amoA* gene was detected using primers specific for *amoA.* These results indicate that, while present, the abundance of the *amoA* gene is low at the time of sampling.

In contrast, Jiang et al. [19] found that the microbial community in Qinghai lake was dominated by AOA and AOB. This discrepancy is likely because the Jiang et al., samples were collected in early summer (July 2005 and 2007). The lake is frozen during the winter and the ice cover causes stratification of the water column, resulting in gradients of salinity, light availability, and oxygen. In spring, inflow glacial meltwater and thawing permafrost reduces the lake salinity and increases the flow of organic matter and nutrients into the lake [10], . The early summer samples had a DO concentration of 5.4 ppm at the surface and 7 ppm at the bottom, salinity was 1.25%, and the ammonia concentration was 5.6 mM. High ammonia concentrations in early spring water are conducive for AOA and AOB. Late summer samples described here (late August 2011) had higher DO (8.9 ppm), higher salinity (1.4%), and little measureable ammonia. This suggests that, similar to many inland lakes and oceans [53]–[56], seasonal changes in the physicochemical conditions and microbiology occurs in Qinghai Lake.

### Comparison of sites B and E

An additional 100 genera were detected in the B_DNA sample compared to the E_DNA sample. Both sites had similar rarefaction curve asymptotes (Figure S1), suggesting that the difference in genera is likely because more reads were obtained for the B_DNA sample (∼6 million more reads) (Table 2). This also influenced the number of ORFs and protein features detected between the two sites (Table 2). Sites B and E were very similar in the taxonomy and functional genes that were detected; however, slight differences were observed in some RNA:DNA ratios. For example, differences were observed in RNA:DNA ratios of nitric oxide reductase and nitrous oxide reductase. It is difficult to conclude if this is biological or a sampling artifact. Small changes in the RNA reads annotated to these genes could translate to large RNA:DNA ratio changes. For example, nitrous oxide reductase at site E had nine DNA reads annotated and would only need four RNA reads to obtain a RNA:DNA ratio similar to site B. Furthermore, site E had less photosynthetic activity than site B. This is more likely a biological phenomenon because of the high abundance of reads annotated to photosystem II and I. While both sites had similar physicochemical conditions (Table 1), a possible reason for this difference is that site E was 1 m deeper than site B and as a consequence receives less photosynthetically active radiation (PAR) [7] because of the attenuation of PAR in water [47].

### Biodiversity

**Figure 6:**
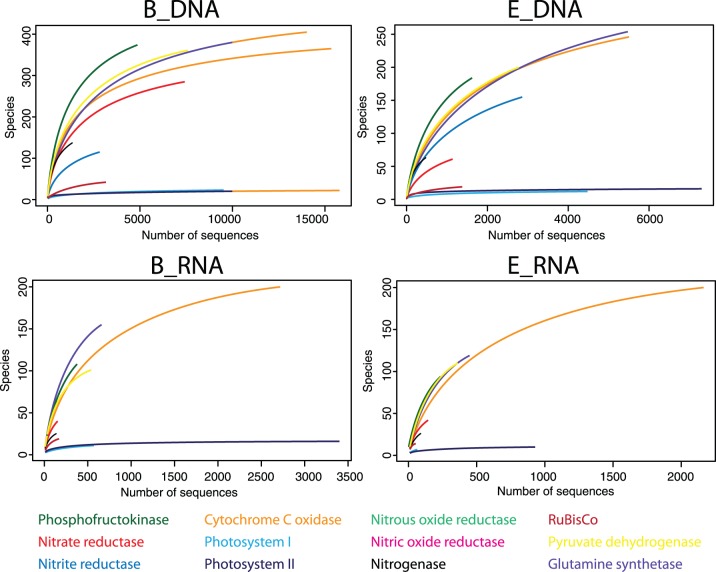
Rarefaction curves for the detected genes related to carbon and nitrogen cycle. Highly saturated curves (e.g. photosystem I and II) indicate that sequencing depth was sufficient to capture most species containing the corresponding gene.

The ability to assign both taxonomy and function to a sequence allowed for identification of the number of organisms that contained a particular functional gene. The richness of each functional gene was calculated as the number of taxa in which the gene was identified. The diversity was sufficiently sampled to detect the majority of organisms that contained a functional gene involved in the carbon and nitrogen cycle (Figure 6). The most diverse functional genes were genes that were part of the heterotrophic pathway: cytochrome C oxidase, glutamine synthetase, phosphofructokinase, and pyruvate dehydrogenase; while photosystem I, photosystem II, and genes involved in the denitrification process had low diversity (Figure 6).

Generally, there is a positive relationship between ecosystem function and taxonomic diversity, because a more diverse community increases the likelihood of finding high-performance key species or the probability of functional redundancy [57], [58]. For example, in soils with a high taxonomic diversity, denitrification rates were higher and decreased more slowly with increased salinity [6]. Similar results were found for respiration [59]–[61], photosynthesis [62]–[64], and nitrogen fixation [65]. This suggests that, as a consequence of the greater diversity, the heterotrophic pathway in Qinghai Lake would be more resilient to stress.

**Figure 7:**
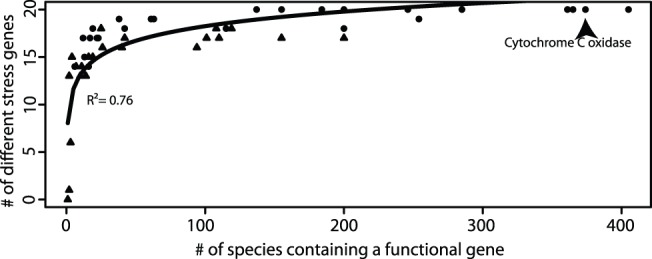
Plot showing the number of species that contained a particular functional gene in the carbon and nitrogen cycle and the number of stress genes that were found in all species with that functional gene (Cytochrome C oxidase is labeled as an example). Triangle symbols represent RNA samples and circle symbols represent DNA samples. Best-fit curve followed a logarithmic function.

While the greater diversity increases the probability of stress resilience, a mechanism to respond to stress is needed to continue functioning. Stress response genes are one mechanism that can be detected with metagenomics and metatranscriptomics. Genes were found that respond to UV, osmotic, and oxidative stress (Figure 4). The detection of these stress genes in Qinghai Lake was expected because of the intense UV radiation and oxidative stress (related to the high elevation of the lake) and its salinity. In addition, samples were retrieved mid-day when UV radiation was at its highest. Furthermore, the number of stress gene types increased logarithmically with the species richness for each functional gene involved in the carbon and nitrogen cycles (Figure 7). For example, in the 365 species that had a cytochrome c oxidase, there were a total of 20 different stress response genes. This observation provides a possible mechanism as to why higher biodiversity is more resilient to stress because one of the species will be able to respond to a stress.

## Conclusions

A metagenomic and metatranscriptomic survey of two locations in Qinghai Lake identified microbial processes involved in the carbon and nitrogen cycle. While photoautotrophic organisms were the most abundant in the DNA samples, heterotrophic organisms were the most active at both sites. Energy generation was also supplemented by photoheterotrophy. Coupled with previous reports from early summer sampling, our data suggest that successional changes occur in the microbiota throughout the summer. Our data also shows a positive relationship between the number of stress gene types and the species richness for each functional gene in the carbon and nitrogen cycles, suggesting that microbial processes with higher taxonomic diversity would have an increased ability to respond to a variety of stresses. Heterotrophic respiration and ammonia assimilation had the highest richness and would be expected to be more resistant to environmental change.

## Supporting Information

Figure S1 **Rarefaction curves showing the number of genera that are detected with the increased number of sequences for the DNA of sites B and E.**
(EPS)
