# Correction: Taxonomic and Functional Diversity Provides Insight into Microbial Pathways and Stress Responses in the Saline Qinghai Lake, China

**DOI:** 10.1371/journal.pone.0116444

**Published:** 2014-12-23

**Authors:** The PLOS ONE Staff

An error was introduced during the typesetting process. Figure 3 is incorrect. Please see the correct Figure 3 and its legend here. The publisher apologizes for the error.

**Figure 3:**
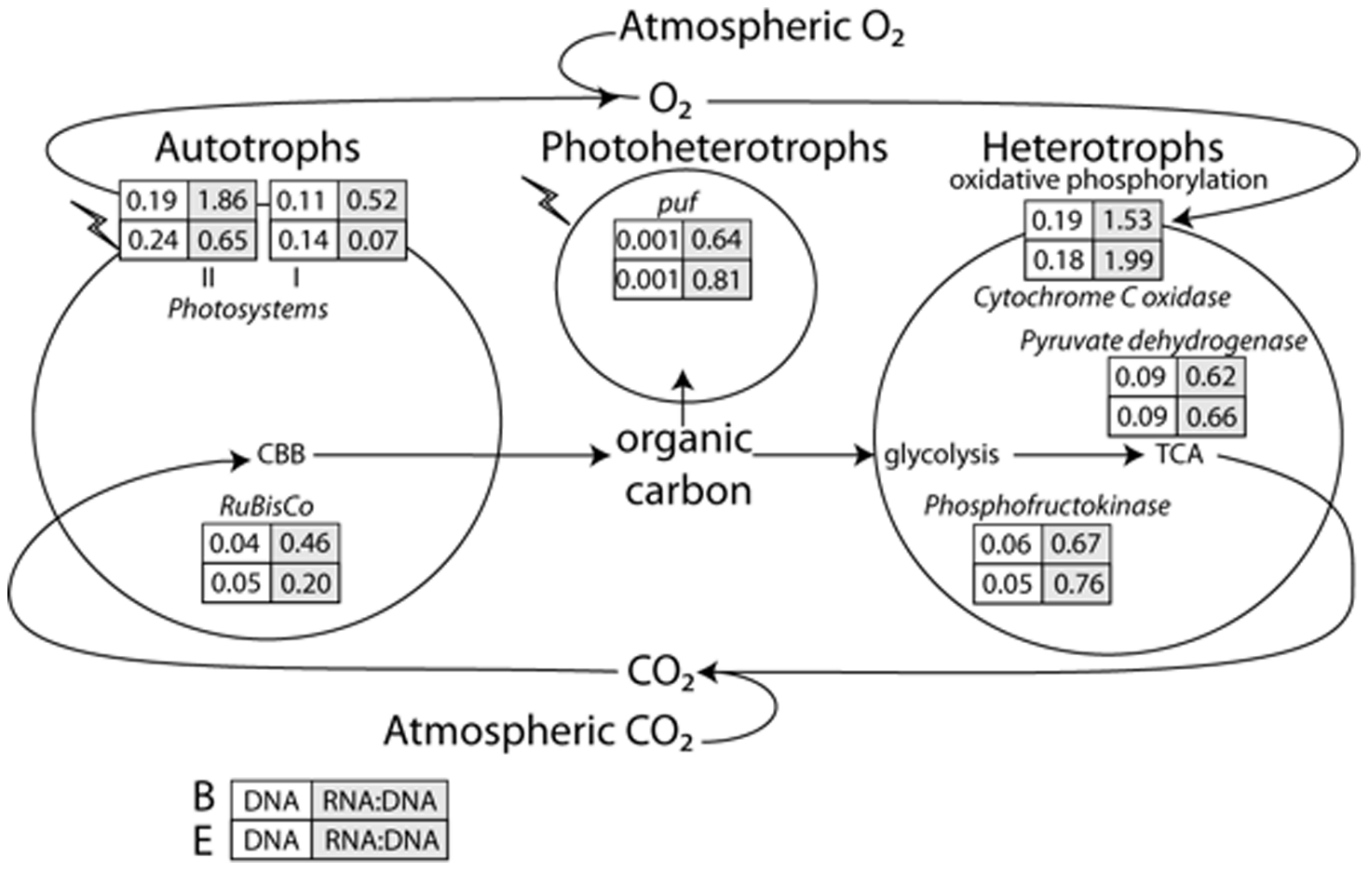
The carbon cycle depicted by a generalized autotroph, photohetertroph, and heterotroph in Qinghai Lake. The numbers in boxes represent the percentage and the RNA:DNA ratio of reads that were annotated within each metabolic pathway for sites B and E. The key genes used to identify a pathway was Ribulose-bisphosphate carboxylase (RuBisCo): Calvin-Benson-Bassham cycle (CBB), D-glucose 6-phosphotransferase: glycolysis, pyruvate dehydrogenase: tricarboxylic acid cycle (TCA), and cytochrome C oxidase: oxidative phosphorylation.
